# Editorial Note: An assessment of optimizing biofuel yield percentage using K-fold integrated machine learning models for a sustainable future

**DOI:** 10.1371/journal.pone.0339811

**Published:** 2026-01-06

**Authors:** The PLOS One Editors

The *PLOS One* Editors issue this Editorial Note to inform readers of concerns regarding compliance with PLOS Authorship policy for this article [1]. We regret that the issues were not addressed prior to the article’s publication.
